# Supplementary material for: Insights into the novel Enterococcus faecalis phage: A comprehensive genome analysis
Source: PLoS One. 2024 May 14;19(5):e0301292. doi: 10.1371/journal.pone.0301292 (PMC11093359; doi:10.1371/journal.pone.0301292)
Supplement: Attachment — Submitted filename: minor revision.docx [file pone.0301292.s001.docx]

Insights into the Novel *Enterococcus faecalis* Phage: A Comprehensive Genome Analysis –

**The overall work is good. Methods and materials results and discussions are represented clearly.**

**I encourage the authors to revise their manuscript and to resubmit it to the journal.**

**Give the explanation regarding the following comments:**

1. In discussion part please mention supporting results of previous study.
2. Why you only choose *Enterobacter faecalis* instead of *E. coli?* Is there any special characteristics having *Enterobacter faecalis*? Explain clearly in discussion part.

The overall paper is good. I strongly agree this work as a publication.
